# Cone-Beam Computed Tomography-Guided Adaptive Radiotherapy: A Case Report on Dynamic Tumor Response in Lung Adenocarcinoma

**DOI:** 10.7759/cureus.66746

**Published:** 2024-08-13

**Authors:** Tinatin Alaverdashvili, Mikheil Baramia

**Affiliations:** 1 Radiation Oncology, Todua Clinic, Tbilisi, GEO

**Keywords:** adaptive radiation therapy, precision radiation therapy, treatment replanning, cone beam computed tomography (cbct), lung adenocarcinoma, radiation therapy

## Abstract

Cone-beam computed tomography (CBCT) is an essential tool in radiotherapy, enhancing patient positioning accuracy and enabling precise treatment delivery by monitoring anatomical changes throughout the treatment process. This case report highlights the significant role of CBCT in managing a patient with lung adenocarcinoma treated with concurrent chemoradiation. The lung mass and lower paratracheal lymph nodes were irradiated with 60 Gy in 30 fractions. During the course of treatment, CBCT allowed us to observe substantial tumor shrinkage, prompting a treatment replanning to ensure optimal targeting of the tumor while minimizing radiation exposure to healthy tissues. This adaptive approach resulted in excellent treatment outcomes with no complications, demonstrating the efficacy of CBCT in modern radiotherapy.

## Introduction

Lung cancer accounts for 12.4% of all cancer diagnoses worldwide, making it the most frequently diagnosed cancer. It is also the most common cause of cancer death, with an estimated 1.6 million deaths each year. Around 85% of lung cancers are non-small cell lung cancer (NSCLC), with adenocarcinoma being the most common subtype [1,2].

Approximately 30-40% of patients present with locally advanced disease where surgical resection is often not feasible. The standard treatment for patients with good performance status and unresectable, locally advanced NSCLC involves definitive chemoradiation therapy. This approach delivers radiation doses ranging from 60 to 66 Gy, administered in daily fractions of 1.8 to 2.0 Gy, concurrently with chemotherapy regimens such as carboplatin/paclitaxel or cisplatin/etoposide [3].

Accurate tumor targeting and dose delivery are essential to achieving optimal therapeutic outcomes for lung cancer patients while minimizing damage to surrounding healthy tissues. In modern external beam image-guided radiotherapy (IGRT), cone-beam computed tomography (CBCT) is essential for ensuring precise patient positioning. Moreover, CBCT supports adaptive radiotherapy (ART) by displaying daily anatomical variations, eliminating the need for additional computed tomography (CT) rescans [4].

This case report highlights the integration of adaptive radiotherapy through the use of daily CBCT imaging, which enabled real-time monitoring of tumor changes and prompt treatment replanning.

## Case presentation

A 55-year-old female patient reported several episodes of hemoptysis over the past few weeks, along with shortness of breath and fatigue. She has a history of arterial hypertension, which is controlled with medications. The patient is a non-smoker and has no family history of cancer.

**Figure 1:**
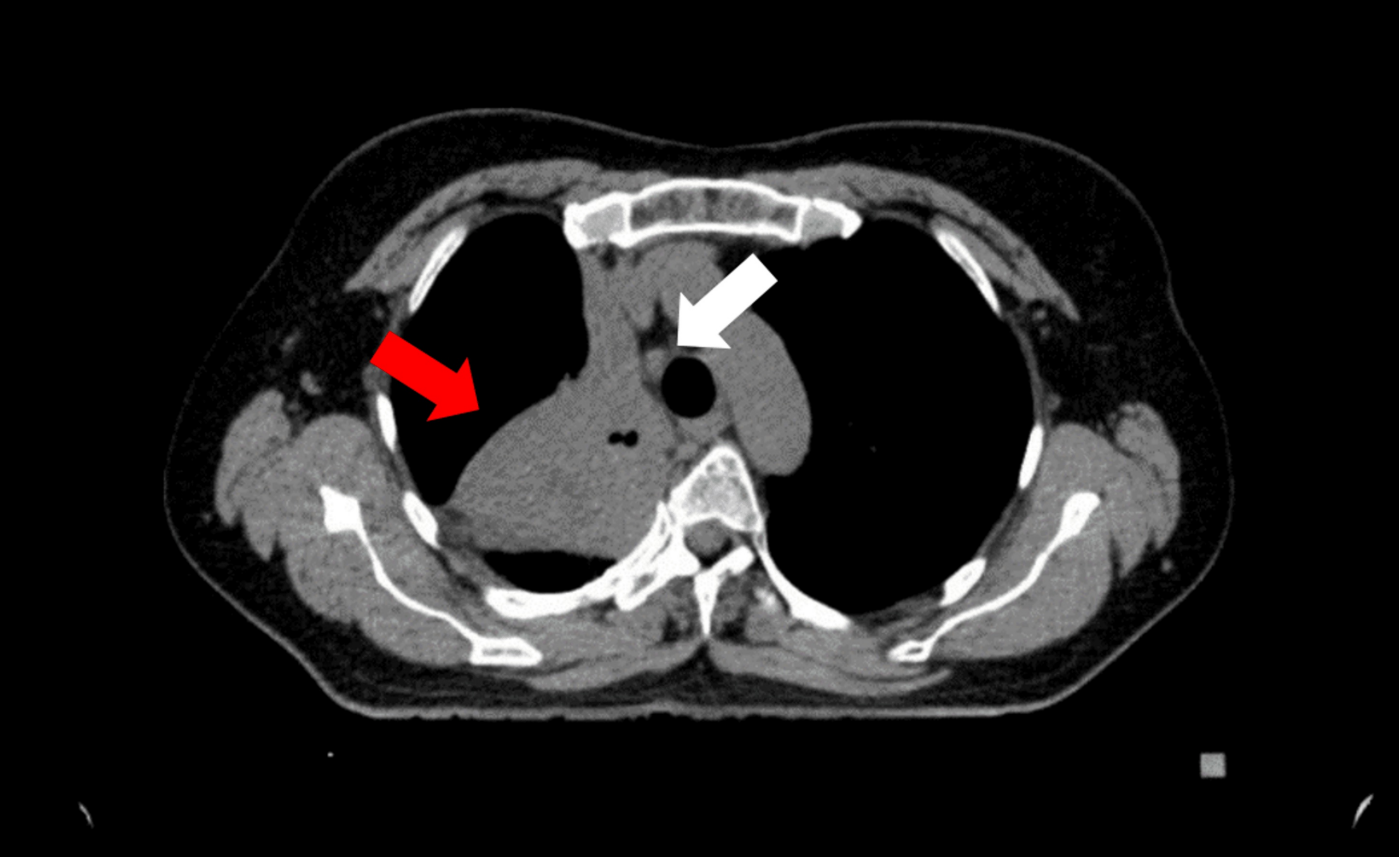
Chest CT image showing the tumor (indicated by the red arrow) and the paratracheal lymph node (indicated by the white arrow)

A chest CT revealed a mass in the apical part of the right lung. The mass, measuring 62 x 52 x 40 mm, was located adjacent to the costal and mediastinal pleura, causing obstruction of the upper and middle bronchi. The scan also identified mediastinal lymphadenopathy. The largest lymph node, located in the upper mediastinum, measured up to 11 mm in diameter (Figure 1).

**Figure 2:**
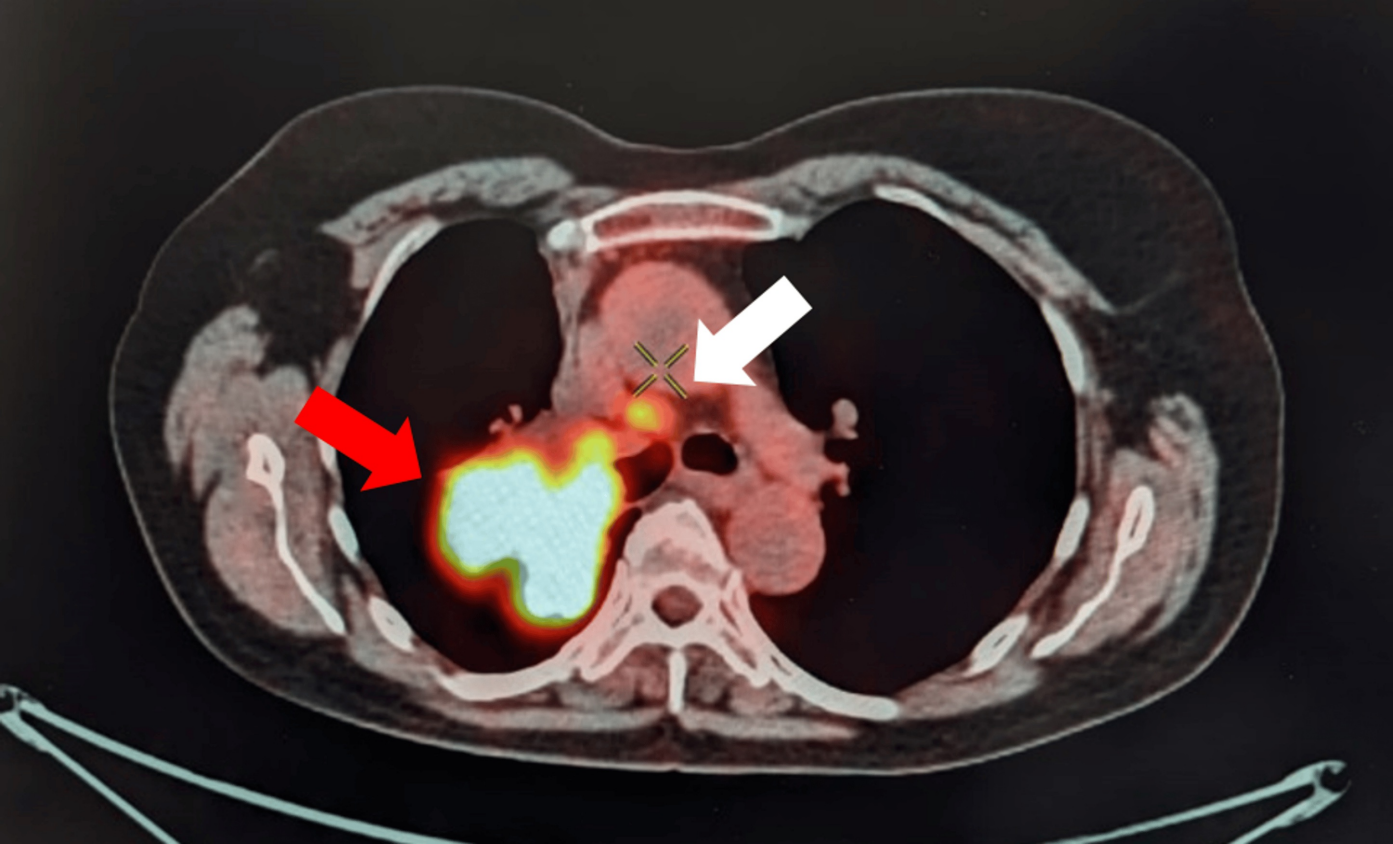
PET-CT image showing a hypermetabolic mass in the right lung (indicated by the red arrow) and a hypermetabolic lower paratracheal lymph node (indicated by the white arrow) PET-CT: positron emission tomography CT

A bronchoscopy with biopsy was performed, and the pathological examination confirmed adenocarcinoma. Brain magnetic resonance imaging (MRI) with contrast and positron emission tomography CT (PET-CT) were also performed. The brain MRI showed no abnormalities or metastases. The PET-CT scan revealed a hypermetabolic mass in the right lung, measuring 64 x 63 x 62 mm with a maximum standard uptake value (SUVmax) of 19. Additionally, there was hypermetabolic lymphadenopathy involving the right lower paratracheal region characterized by an SUVmax of 8 (Figure 2). The tumor was classified as cT3N2M0 (stage III B).

Radiation therapy was planned concurrently with carboplatin and paclitaxel. On a CT simulation, the patient was placed in a supine position with arms above the head and without immobilization. The CT scan was performed with a slice thickness of 3 mm. For more precise treatment and less exposure to healthy tissues, we employed the deep inspiration breath hold (DIBH) technique. The patient practiced breath holds during the simulation to ensure she could maintain consistent and reproducible breath-hold durations. A respiratory monitoring device was used to track the breath-hold position and provide real-time feedback. The patient was instructed to hold her breath at specific time intervals, typically 15-20 seconds, which she did successfully, allowing for clear imaging and accurate treatment planning.

After conducting CT simulation, we delineated target volumes and identified organs at risk (OARs). The gross tumor volume (GTV) was registered by co-registering the CT simulation images with the PET-CT images, utilizing the metabolic activity observed in the PET-CT to accurately outline the tumor extent. This co-registration process ensured that the high metabolic areas indicative of the tumor were precisely marked on the CT images. In our case, the GTV included the right lung mass and the right lower paratracheal lymph nodes, both of which were metabolically active on the PET-CT. The clinical target volume (CTV) encompassed the GTV, right lung mass, and right lower paratracheal lymph nodes. A 4-mm expansion from the CTV was used to define the planning target volume (PTV). OARs included the lungs, trachea, main bronchus, esophagus, spinal cord, heart, great vessels, pulmonary artery, and aorta. Treatment was delivered using 6 MV photons on the TrueBeam LINAC (Varian Medical Systems, Palo Alto, CA). A total dose of 60 Gy was delivered to the right lung mass and right lower paratracheal lymph nodes, with a daily dose of 2 Gy in 30 fractions using the deep inspiration breath hold.

Daily CBCT scans were employed to ensure precise radiation administration. The CBCT images were registered to the planning CT using a combination of fixed anatomical landmarks and soft tissue alignment. The primary focus was on aligning the bony structures, such as the vertebrae and ribs, to ensure consistent positioning. Additionally, soft tissue matching was performed to verify the alignment of the GTV, ensuring that the tumor and adjacent structures were accurately targeted. This dual approach helped maintain the precision of the treatment throughout the course of therapy.

The patient developed a subfebrile temperature starting from the 12th fraction of radiation therapy. After completing 15 fractions, she presented to our department with a fever of up to 39 degrees Celsius. She was promptly referred to an infectious disease specialist. Laboratory tests revealed elevated C-reactive protein levels and appropriate antimicrobial therapy was initiated. Radiation therapy was paused that day. By the following day, the patient showed significant improvement and radiation therapy resumed without further complications observed during or after treatment.

**Figure 3:**
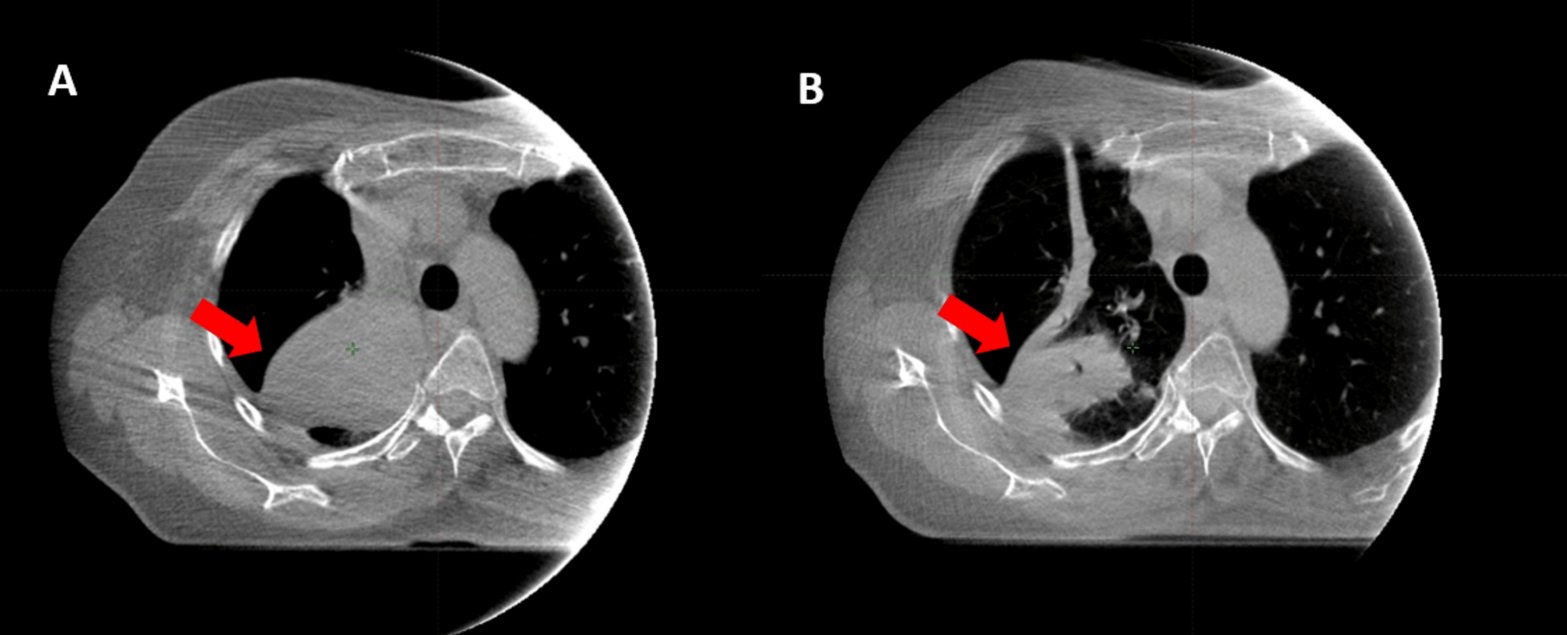
Comparison of tumor size and shape (indicated by the red arrow) on the first CBCT (A) and the 21st CBCT (B) CBCT: Cone-beam computed tomography

During treatment, a CBCT image taken on the 21st fraction day revealed a significant shrinkage of the mass, with a reduction in the GTV by approximately 38.7%. The initial GTV, measured at 67.5 cm³, decreased to 41.4 cm³ (Figure 3). These changes in the targeted volumes necessitated treatment replanning.

**Figure 4:**
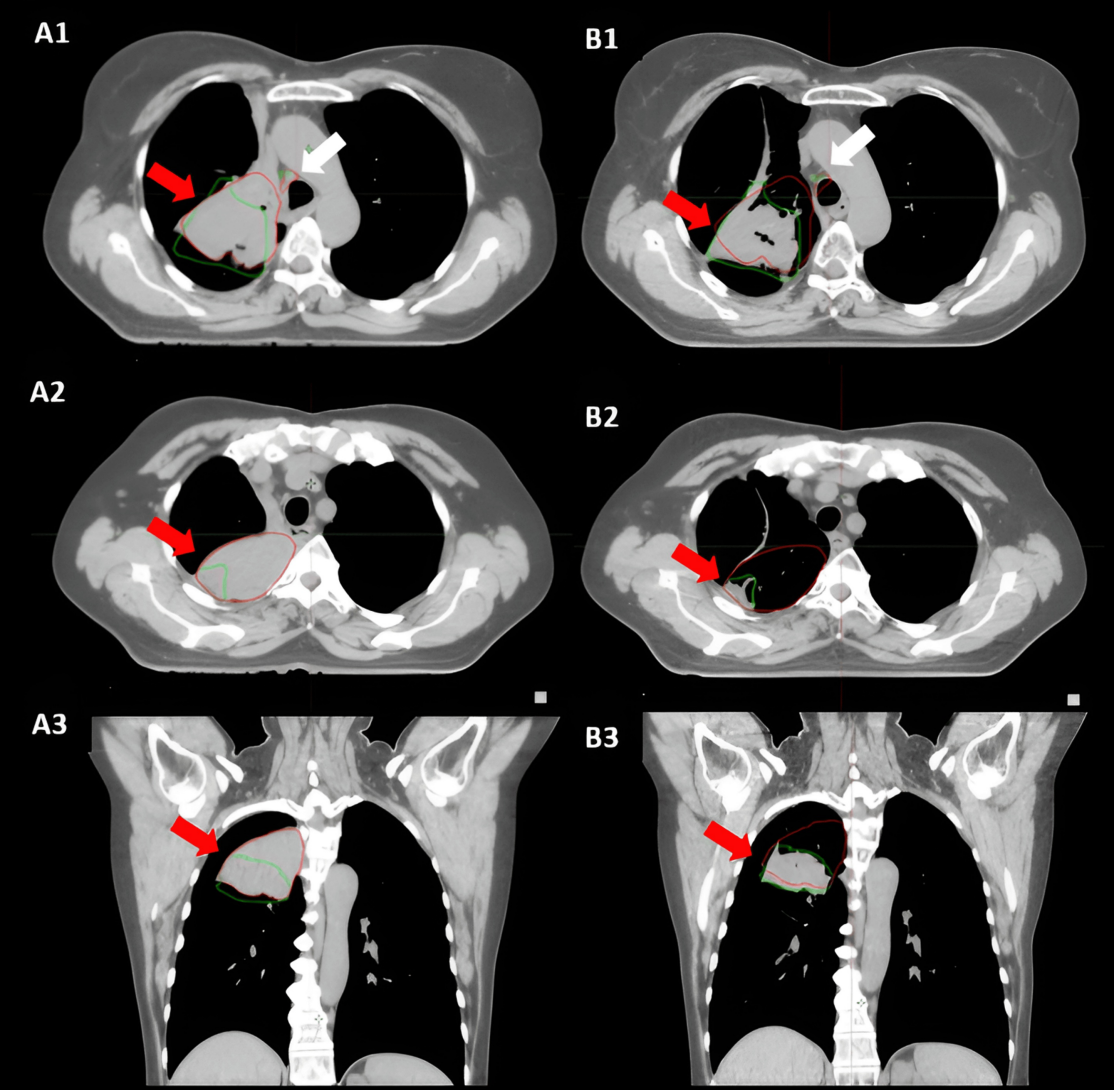
Comparison of gross tumor volumes (GTVs) from fused simulation CT scans A1, A2, A3 – first CT simulation. B1, B2, B3 – re-simulation. GTVs from the first treatment plan are contoured in red, and GTVs from the re-simulation are contoured in green. Red arrows indicate the tumor and white arrows indicate the paratracheal lymph node.

Subsequently, we performed a CT re-simulation and developed a new treatment plan (Figure 4). Dosimetric data of the initial treatment plan and the re-planned treatment are presented in Table 1. The replanning allowed for more precise targeting of the reduced tumor volume while sparing the surrounding healthy tissues. By updating the PTV to reflect the decreased GTV, the new plan minimized the radiation dose to the organs at risk. The improved conformity of the radiation dose to the shrunken tumor volume reduced the risk of radiation-induced toxicity and potentially enhanced the overall treatment efficacy. Additionally, the revised plan ensured that the remaining tumor received the prescribed dose of 60 Gy, maintaining the treatment's therapeutic goals. The patient received the last nine fractions of radiation therapy according to the new plan, starting the day after the re-simulation.

**Table 1:** Dosimetric data of the initial and adaptive plans GTV: gross tumor volume; CTV: clinical target volume; PTV: planning target volume; OAR: organs at risk

Parameters	Initial Treatment Plan	Adaptive Treatment Plan
Target Volumes		
GTV (cc)	67.5	41.4
CTV (cc)	80.0	60.0
PTV (cc)	100.0	75.0
Dose Prescription		
Total Dose (Gy)	60	60
Fractionation Schedule	30 x 2 Gy	30 x 2 Gy
Dosimetric Parameters		
GTV D95% (Gy)	57	59
GTV D98% (Gy)	55	57
GTV Dmax (Gy)	65	63
CTV D95% (Gy)	58	59
CTV Dmax (Gy)	66	65
PTV D95% (Gy)	58	59
PTV Dmax (Gy)	66	65
OAR		
Lung V20 (%)	25	20
Lung mean dose (Gy)	12	10
Heart Dmax (Gy)	35	32
Heart mean dose (Gy)	8	6
Spinal cord Dmax (Gy)	45	43

**Figure 5:**
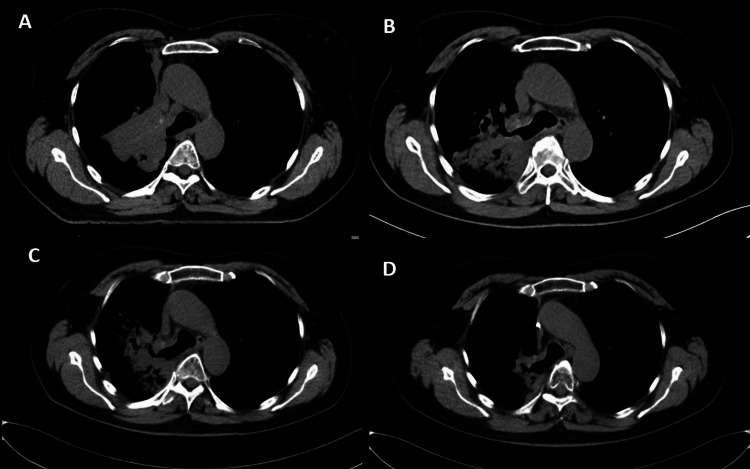
Chest CT scans before (A) and after (B,C,D) radiation therapy (A) Before treatment; (B) two months after RT; (C) four months after RT; (D) 16 months after RT

Two months after completing radiation therapy, a CT scan of the chest indicated a positive response: the lung mass measured 34 mm in diameter (reduced from 64 mm before RT), and the largest lower paratracheal lymph node measured 10 mm (reduced from 11 mm before RT). At four months post-treatment, the CT scan showed further reduction in the lung mass to 21 mm in diameter, with the lower paratracheal lymph node decreasing to 8 mm. Sixteen months post-treatment, the CT scan revealed no focal infiltrative changes or lymphadenopathy (Figure 5). The patient did not undergo follow-up CT scans between these intervals.

## Discussion

The management of locally advanced NSCLC represents a significant clinical challenge due to the tumor's proximity to critical structures and potential for rapid growth and metastasis. For patients like ours, with unresectable disease, concurrent chemoradiation remains the standard of care, aiming to achieve local tumor control while minimizing systemic toxicity.

In nearly 40% of cases, conventional RT irradiates a significant amount of healthy tissue surrounding the tumor, leading to radiation-induced lung injury (RILI), which includes radiation pneumonitis (RP) and radiation fibrosis (RF). To mitigate these risks, new treatment delivery technologies such as intensity-modulated RT (IMRT), volumetric arc radiotherapy (VMAT), and stereotactic body radiation therapy (SBRT) have been developed. These advancements enable highly individualized radiation treatment for primary tumors, aiming to minimize lung injury [5]. The integration of CBCT with DIBH enhances treatment precision, further reducing the potential for lung damage.

In this case report, CBCT played a pivotal role in our treatment strategy. Providing daily imaging, CBCT allowed us to adapt our radiation therapy plan in response to observed anatomical changes, particularly significant tumor shrinkage. This adaptive approach is crucial in modern radiotherapy, where precise tumor targeting is essential for maximizing therapeutic efficacy and minimizing radiation-induced toxicity to adjacent healthy tissues.

Our patient, a 55-year-old female with stage III B lung adenocarcinoma, received a total dose of 60 Gy in 30 fractions, concurrently with carboplatin/paclitaxel chemotherapy. The use of the DIBH technique enhanced treatment safety. The major benefit of DIBH for lung cancer radiotherapy is the changed anatomy that enables dose reduction compared with free breathing through larger lung volume and increased distance between the tumor and the heart [6]. However, during the treatment course, she experienced a brief interruption due to fever, which resolved promptly with medical management, highlighting the importance of comprehensive patient care in managing treatment-related complications.

Observing significant change in target volume during treatment required replanning after 21 fractions of radiation therapy. The implementation of adaptive replanning without any treatment delay optimized our radiation therapy strategy, ensuring accurate dose delivery to the revised target volumes. This adaptive approach provided several dosimetric advantages that significantly improved the treatment's efficacy and safety profile.

One key benefit of adaptive replanning was the ability to update the PTV to accurately reflect the reduced GTV. This adjustment led to better conformity of the radiation dose to the smaller tumor size, allowing us to deliver a higher therapeutic dose while minimizing exposure to surrounding healthy tissues. The updated plan ensured that both the revised GTV and CTV received the prescribed dose of 60 Gy, maintaining the treatment’s therapeutic efficacy.

Additionally, the replanning process reduced the radiation exposure to nearby OARs, such as the lungs, esophagus, and heart. This reduction minimized the potential for radiation-induced toxicity and side effects, contributing to a better overall treatment experience for the patient.

By tailoring the radiation plan to the updated tumor size, the adaptive strategy significantly increased the likelihood of effective tumor control, ultimately leading to improved treatment outcomes. This was confirmed by subsequent imaging evaluations conducted at two, four, and sixteen months post-treatment, which demonstrated a positive treatment response characterized by a substantial reduction in tumor size and no evidence of new disease progression.

## Conclusions

This case underscores the critical role of advanced radiotherapy techniques, including CBCT-guided adaptive planning and the use of DIBH, in effectively managing stage III B lung adenocarcinoma. These approaches not only facilitated precise tumor targeting and minimized radiation-induced toxicity but also led to significant treatment response and favorable clinical outcomes. The successful integration of these technologies highlights their potential to enhance therapeutic efficacy while improving patient safety in the treatment of locally advanced lung cancer.

## References

[1] Bray F, Laversanne M, Sung H, Ferlay J, Siegel RL, Soerjomataram I, Jemal A (2024). Global cancer statistics 2022: GLOBOCAN estimates of incidence and mortality worldwide for 36 cancers in 185 countries. CA Cancer J Clin.

[2] Herbst RS, Morgensztern D, Boshoff C (2018). The biology and management of non-small cell lung cancer. Nature.

[3] Wald P, Mo X, Barney C (2017). Prognostic value of primary tumor volume changes on kV-CBCT during definitive chemoradiotherapy for stage III non-small cell lung cancer. J Thorac Oncol.

[4] Maspero M, Houweling AC, Savenije MH, van Heijst TC, Verhoeff JJ, Kotte AN, van den Berg CA (2020). A single neural network for cone-beam computed tomography-based radiotherapy of head-and-neck, lung and breast cancer. Phys Imaging Radiat Oncol.

[5] Arroyo-Hernández M, Maldonado F, Lozano-Ruiz F, Muñoz-Montaño W, Nuñez-Baez M, Arrieta O (2021). Radiation-induced lung injury: current evidence. BMC Pulm Med.

[6] Josipovic M, Aznar MC, Thomsen JB (2019). Deep inspiration breath hold in locally advanced lung cancer radiotherapy: validation of intrafractional geometric uncertainties in the INHALE trial. Br J Radiol.

